# Effects of Socioeconomic Status on Physical and Psychological Health: Lifestyle as a Mediator

**DOI:** 10.3390/ijerph16020281

**Published:** 2019-01-20

**Authors:** Jian Wang, Liuna Geng

**Affiliations:** School of Social and Behavioral Sciences, Nanjing University, Nanjing 210023, China; dr.jianwang@smail.nju.edu.cn

**Keywords:** socioeconomic status, lifestyle, physical health, psychological health

## Abstract

Health is correlated to people’s socioeconomic status (SES) and lifestyle. This study examined the impact of SES on respondents’ physical and psychological health. Moreover, we explored the potential mediating effect of lifestyle on the relationship between SES and health. The participants were 986 respondents from the 2015 Chinese General Social Survey (CGSS). Structural equation modeling (SEM) was used to test the hypothesized relationship between the variables. The results indicated that SES had a significant impact on people’s physical health, but the impact of SES on psychological health was not significant. Lifestyle had significant positive effects on both physical and psychological health. In addition, lifestyle mediated the relationship between SES and health. This research is helpful in gaining a better understanding of the relationship and mediating mechanism between SES, lifestyle, and health. It is recommended that research with longitudinal design and comprehensive indicators be undertaken in the future.

## 1. Introduction

The relationship between socioeconomic status (SES)and health has been studied for a long time. In the 1960s, academics generally believed that with medical technology and economic development, health inequality would be reduced, at least in developed countries [1]. However, in the 1980s, Black found that health inequality in Britain has not only not diminished, but actually increased [2]. Studies in the United States and European countries have also supported this conclusion, that is, the health condition of the group with higher SES is obviously better than that of the group with lower SES [3,4]. These studies confirmed the profound impact of SES on health; the mechanism behind this phenomenon, however, has been a matter of debated. Scholars have proposed two different perspectives: social causation theory and health selective theory [5]. The former suggests that the difference in SES is the root cause of health inequalities [6]. On the contrary, the latter implies that people with good health tend to move upward and thus have a higher SES [7]. Despite these arguments, there appears to be a growing agreement that the influence of SES on health is closely related to people’s lifestyle [8]. In a more concrete context, health is maintained and improved through the efforts and healthy lifestyle choices of individuals [9].

A healthy lifestyle refers to a series of behavioral patterns through which individuals maintain and promote good health based on certain motivations, norms, abilities, and knowledge about what constitutes healthy, stress relieving, or pleasurable behaviors [10]. Lifestyle involves both health risk behaviors, such as smoking, drinking, and sedentariness [11,12] and health-promoting behaviors, such as physical exercise, interpersonal interaction, stress management, and spiritual growth [13]. Research findings have indicated that health is closely related to people’s lifestyles in a wide range of social contexts. For example, an investigation conducted in the United States showed that the actual leading causes of mortality are behavioral risk factors, such as smoking, poor diet, and physical inactivity. The study concluded that lifestyle-related behavioral factors account for nearly 40% of deaths [14]. Moreover, lifestyles could be passed down across generations. A study of mother-child pairs suggested that if a mother of a 0–3 years old child has a healthy lifestyle, the child is 27% more likely to be healthy and adopt the same lifestyle [15]. In addition to physical health, lifestyle has also been noted to be associated with psychological health. Persons with unhealthy lifestyles usually have worse mental health compared to those with healthy lifestyles. For instance, risk behaviors, such as smoking, have been reported to be associated with poor mental health [16]; by contrast, health-promoting behavior, such as taking physical exercise, have been recommended as an effective intervention to relieve depression [17]. Recently, a large social survey targeting New Zealand adults revealed that people who adopt a healthier lifestyle are more likely to have optimal well-being [18]. Notably, although lifestyle is closely related to one’s health, it is not purely a personal choice. In fact, lifestyle is influenced by a variety of social factors, especially an individual’s SES.

Lifestyle and SES are strongly related. As Max Weber noted, one can choose a certain lifestyle from the existing choices, but the range of possible choices is largely determined by one’s SES and other social determinants [19]. Similarly, according to Cockerham, lifestyle is fundamentally constrained by social hierarchy and an individual’s living conditions [20]. Moreover, a study indicated that excluding the effect of lifestyle from the overall effect of socioeconomic status on health would significantly reduce the latter [21]. Therefore, lifestyle could be one of the intermediate mechanisms linking SES and health. It is noteworthy that although the literature suggests a strong correlation between SES, lifestyle, and health, the nature of their relationship is not well-specified. On one hand, previous studies routinely focused on single behavior or small subsets of lifestyle behaviors (especially risk behaviors, such as drinking and smoking), but focusing on single or small subsets of behaviors offers limited implications [22]. On the other hand, many of the existing studies on SES and lifestyle have concentrated on people’s physical health [16]. However, the modern construction of health includes both physical and psychological health.

**Figure 1:**
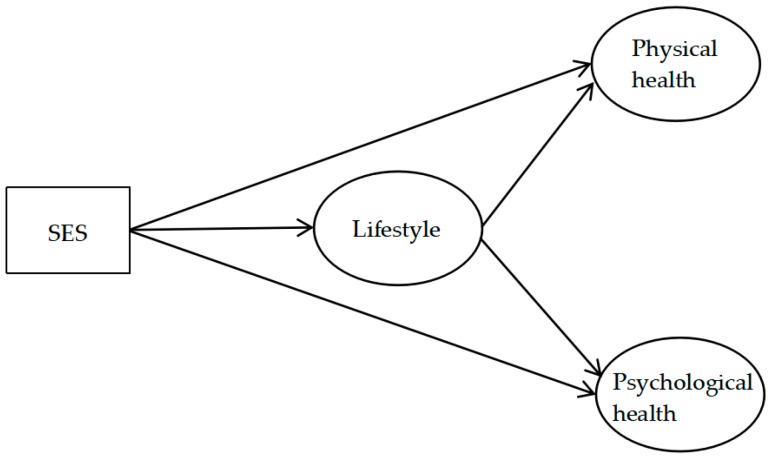
Conceptual framework.

The current study used national social survey data to investigate the relationship between SES and health, and explored the possible mediating role of lifestyle. Specifically, we adopted International Socio-Economic Index (ISEI) as the indicator of SES, and several health-promoting behaviors as the indicators of lifestyle. Furthermore, this study considered a broad concept of health which involves not only physical health but also psychological health. The hypotheses and conceptual framework (Figure 1) of this study are as follows:

H1a: People with higher SES are in better physical health.
H1b: People with higher SES are in better psychological health.
H2a: People with a healthier lifestyle are in better physical health.
H2b: People with a healthier lifestyle are in better psychological health.
H3a: Lifestyle mediates the relationship between SES and physical health.
H3b: Lifestyle mediates the relationship between SES and psychological health.

## 2. Materials and Methods

### 2.1. Sample

Data used in the current study were obtained from the 2015 Chinese General Social Survey (CGSS), a continuous large-scale nationwide survey conducted by the National Survey Research Center of China. The CGSS 2015 employed a stratified multistage probability proportional to size (PPS) sampling design and covered 478 villages in 28 provinces across mainland China. The CGSS 2015 consists of six modules, with different sample size in each module. In this study, we employed the Core module (sample size 10,968) and the East Asia Social Survey module (sample size 1828). Since 724 people were unemployed, we were unable to calculate the International Socio-Economic Index (ISEI); so these samples were not included in the study. Additionally, 118 invalid questionnaires were also removed, reducing the final sample size to 986. Data are available through the Chinese National Survey Data Archive website [23].

The participants were 532 males (53.95%) and 454 females (46.04%). Their mean age was 44.69 (SD = 13.36). Of the total 986 respondents, 522 live in cities (52.94%) and 464 live in rural areas (47.05%). Further, 800 respondents were married (81.13%) and 186 were single (18.86%). With regard to their educational level, 313 had accomplished primary education level (31.74%), 480 had reached secondary education level (48.68%), and 193 had completed tertiary education (19.57%).

### 2.2. Variables and Instruments

SES was measured by International Socio-Economic Index (ISEI). This index—proposed by Ganzeboom, Graaf, and Treiman in the 1990s [24]—reflects people’s socioeconomic circumstances with respect to education, occupation, and income. According to the standard procedure provided by Ganzeboom and Treiman [25], we converted respondents’ occupation code (isco-88) into ISEI using STATA (Version 15.0, Stata Corp, College Station, TX, USA). ISEI is a continuous variable, with greater value indicating higher SES.

Lifestyle includes both health-risk behaviors and health-promoting behaviors. This study mainly focused on health-promoting behaviors. Lifestyle was measured by a 6-item Likert scale. Respondents were asked whether they often do the following things: (a) “participate in physical exercise”; (b) “attend cultural events, such as concerts, performances, or exhibitions”; (c) “gathering with friends”; (d) “listen to music at home”; (e) “read books, newspapers, or magazines,” and (f) “improve yourself through learning.” The options ranged from 1 (always) to 5 (never). A higher score represents a healthier lifestyle. The Cronbach’s alpha coefficient for the scale was 0.76.

Physical health was measured by participants’ self-reported health. Despite the deviations between self-reported health and people’s objective health condition, self-reported health is still considered a valid indicator of health in various social contexts [26]. In the present study, physical health was assessed by two items: (a) “What do you think of your current physical condition? (1 = very unhealthy to 5 = very healthy)” and (b) “How often have physical health problems affected your work or other daily activities in the past four weeks? (1 = always to 5 = never).” A higher score reflects better physical health. The Cronbach’s alpha coefficient for the scale was 0.78.

Psychological health was measured by a 5-item Likert scale, with response options ranging from 1 (always) to 5 (never): (a) “How often do you feel calm or peaceful?” (b) “How often do you feel cheerful?” (c) “How often do you feel burned out?” (d) “How often do you feel mentally tired?” and (e) “How often do you feel ‘I cannot take it anymore’?” A higher score corresponds to better psychological health. The Cronbach’s alpha coefficient for the scale was 0.75.

Covariates include age, gender (male = 1, female = 2), region (urban areas = 1, rural areas = 2), and marital status (married = 1, single = 2).

### 2.3. Statistical Analysis

Before statistical analysis, the data were screened according to the principle of three standard deviations above or below the mean scores. Missing values were excluded from the analysis. Descriptive statistics and correlation analyses were performed with SPSS (Version 22.0, IBM Corp, Armonk, NY, USA). The structural equation model was tested with Mplus (Version 7.4, Muthén & Muthén, Los Angeles, CA, USA). We tested the mediation structural model with maximum likelihood estimators and 95% bias-corrected confidence intervals (CIs) using 1000 bootstrapped samples. 

The fitness between the current data and the hypothesized model was assessed through the following indicators: (a) the root mean square error of approximation (RMSEA); (b) the standardized root mean square residual (SRMR); (c) the comparative fit index (CFI), and (d) the Tucker-Lewis index (TLI). The RMSEA and SRMR with values below 0.05, and CFI and TLI with values over 0.9, indicate a good fit [27]. 

## 3. Results

### 3.1. Preliminary Analysis

The descriptive statistics and correlation coefficients for the study variables are presented in Table 1. Correlation analyses indicated that SES was significantly correlated with both physical health (*r* = 0.27, *p* < 0.01) and psychological health (*r* = 0.12, *p* < 0.01). Moreover, lifestyle was significantly associated with physical health (*r* = 0.29, *p* < 0.01) and psychological health (*r* = 0.21, *p* < 0.01). In addition, the correlation between SES and lifestyle was significant and positive, too (*r* = 0.56, *p* < 0.01). These results provided good preliminary support for the hypotheses.

### 3.2. Testing the Study Model

**Figure 2:**
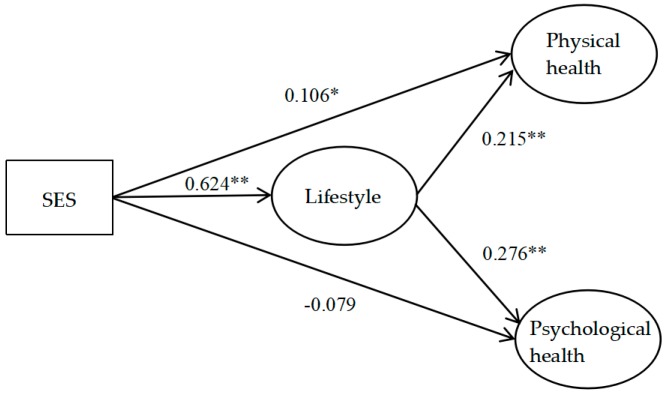
Structural equation model with standardized estimates. Notes: * Significant at level *p* < 0.05, ** Significant at level *p* < 0.01.

The SEM was used to test the proposed study model. The standardized fit indices indicated that the model was appropriate: the RMSEA was 0.046, the SRMR was 0.038, the CFI was 0.941, and the TLI was 0.925. The standardized estimates for the structural model are shown in Figure 2.

The relationships between the variables were examined. The results indicated that SES had a significant positive impact on physical health (*β* = 0.106, *p* < 0.05, 95% CI = [0.016, 0.019]), suggesting that people with higher SES may have better physical health. Hence, H1a was supported. The impact of SES on psychological health, however, was not significant (*β* = −0.079, *p* > 0.05, 95% CI = [−0.179, 0.021]), indicating that people with higher SES may not necessarily be psychologically healthier. Hence, H1b was not supported. Furthermore, lifestyle was demonstrated to have positive effects on both physical health (*β* = 0.215, *p* < 0.01, 95% CI = [0.106, 0.329]) and psychological health (*β* = 0.276, *p* < 0.01, 95% CI = [0.163, 0.389]), implying that people with a healthier lifestyle may be in better physical and psychological health. Thus, H2a and H2b were asserted.

In terms of the mediating effect, it was found that SES had a significant influence on lifestyle (*β* = 0.642, *p* < 0.01, 95% CI = [0.573, 0.674]). Moreover, lifestyle was reported to be a significant mediator in the relationship between SES and physical health (*β* = 0.134, *p* < 0.01, 95% CI = [0.003, 0.011]). Similarly, the results also indicated a significant indirect effect of SES on psychological health, which was mediated by lifestyle (*β* = 0.172, *p* < 0.01, 95% CI = [0.002, 0.005]). Hence, both H3a and H3b were supported.

## 4. Discussion

Using nationally representative data, the current research seeks to contribute to the literature by investigating the impact of SES on health and exploring the mediating effect of lifestyle. Our study established a mediation structural model and yielded three valuable findings. First, SES had a significant positive impact on physical health; but the impact of SES on psychological health was not significant. Second, lifestyle had significant positive effects on both physical and psychological health. Third, lifestyle played a mediating role in the relationship between SES and health. This research is helpful in gaining a better understanding of the relationship between SES, lifestyle, and health.

A substantial body of research has demonstrated the impact of SES on health. Past studies indicated that there is a sustained and stable relationship between SES and health [28]. Some researchers argued that SES may be one of the most decisive social factors that affects an individual’s health and life expectancy [29]. Consistent with existing studies, the results of this study also revealed that SES had a significant impact on physical health. This impact is mainly reflected in three aspects of an individuals’ life: occupation, income, and education. Previous research have found that people with higher professional status enjoy more work autonomy, engage in less manual labor, have fewer occasions of being exposed to health risks. Similarly, higher incomes are usually associated with better nutritional status, housing conditions, medical services, etc. In addition, people with higher levels of education tend to have better health awareness and health-related knowledge [30]. Consequently, higher SES may be correlated with better physical health. Nevertheless, our hypothesis that people with higher SES are in better psychological health was not supported. Compared with physical health, fewer studies have focused on the association between SES and psychological health. Furthermore, the link between SES and mental health remains controversial in previous studies. Some research reported that low SES was associated with the prevalence of psychological distress [31], depression [32], as well as anxiety and anguish [33]. On the contrary, there is evidence implying that mental health has no significant relevance to SES [34]. A more convincing conclusion might be that the relationship between SES and psychological health is different across different mental illnesses [35]. Another possible explanation for our unsupported hypothesis is that people with higher SES have relatively higher job-related stress, and face more psychological stressors [36], which may undermine the health benefits of a high SES. However, this speculation needs to be further studied.

Another finding in this study indicated that lifestyle has a significant influence on both physical and psychological health. This conclusion is consistent with existing studies. Substantial research has demonstrated the correlation between lifestyle and physical health. For instance, lifestyle behaviors (e.g., smoking, drinking, exercise, and sleeping) have been reported to be associated with a variety of diseases, such as obesity [37,38], stroke [39], type II diabetes [40], and cardiovascular diseases [41]. Besides, unhealthy lifestyle behaviors have been listed as the leading causes of premature deaths in many countries [42]. The current study used only one item (exercise) to assess respondents’ physical activity, but the assumption that lifestyle is related to physical health was strongly supported. In addition to physical health, our results also demonstrated that people with a healthier lifestyle are in better psychological health. In general, studies on health and lifestyle have frequently investigated physical health, but psychological health has received limited attention [16]. Despite this, research findings have indicated that lifestyle behaviors were significantly associated with psychological health, such as psychological distress [43], depression [44,45], anxiety [46], and well-being [47]. The present study assessed lifestyle, unlike previous studies, mainly through health-promoting behaviors, and the results also suggested that lifestyle has a significant impact on mental health. Thus, the results add to the evidence base that a healthier lifestyle can improve both physical and psychological health.

The results of the SEM supported our hypotheses that lifestyle mediates the relationship between SES and health. There is ample evidence that SES and lifestyle have a significant impact on people’s health, but the mechanism of this impact lacks sufficient explanation. Social epidemiologists generally regard SES as the distal influencing factor of health, and lifestyle-related behaviors as the proximal factor. However, earlier studies mainly focused on the proximal factor, and overlooked the distal factor [48]. Nevertheless, the impact of macro social structure on people’s health cannot be ignored because people’s lifestyles are largely shaped by their SES [49]. According to the Health Lifestyle Model proposed by Cockerham, lifestyle is not only the result of personal life choices but also of social structural factors (such as social class circumstances, gender, age, race, living conditions, etc.). Under the influence of these two factors, people acquire different behavioral dispositions (habitus), resulting in a series of health practices and behaviors that directly affect an individual’s health [20]. The results of this study were largely consistent with this model, and revealed that persons from lower SES were less likely to report good physical health. At the same time, people with a healthier lifestyle were in better physical and mental health. More importantly, lifestyle might be a mediator of the relationship between SES and health. In other words, SES has direct effects on health through lifestyle behaviors. Lifestyle reflects personal routines that can be characterized as either positive or negative [10]. People with higher SES have stronger motivation and adequate resources to maintain a healthy lifestyle, which further leads to comparative advantages in health.

This research has potential theoretical and practical implications. On one hand, previous research on lifestyle and health was conducted mainly in western countries, the current study, however, provided preliminary empirical evidence from Chinese social context, and found that the trend of health inequality in western countries also exists in China. On the other hand, most of the existing studies only involved physical health. The present study, to some extent, expanded the Health Lifestyle Model [20] by emphasizing relevance of SES and psychological health. Furthermore, the present paper was of certain value in building a systematic evidence-base for interventions and policies to reduce health inequalities [50]. This study highlighted the important impact of lifestyle on health. Thus, a strategy embedding lifestyle intervention in public health promotion programs could be adopted to improve people’s physical as well as mental health.

Some limitations of the present study should be noted. First, although we used structural equation modeling to examine the relationship between variables, owing to the cross-sectional nature of the study, it is difficult to draw causal conclusions. Second, in the dataset used in this study, some respondents were unemployed; so their ISEI could not be calculated, resulting in a large number of missing values. Finally, the lifestyle scale used in the current study mainly focused on health-promoting behaviors; if more behavioral indicators (e.g., smoking, drinking, etc.) were adopted, the results would be more convincing.

## 5. Conclusions

The study examined the impact of SES on health and focused on the mediating role of lifestyle. The findings indicate that SES was significantly associated with physical health, but not with psychological health; lifestyle had significant positive effects on both physical and psychological health; and lifestyle mediated the relationship between SES and health. These findings enhance our understanding of the relationship and the mediating mechanism between SES, lifestyle, and health. It is recommended that research with a longitudinal design and comprehensive indicators be undertaken in the future.

## Figures and Tables

**Table 1:** Descriptive statistics and correlation coefficients for the study variables.

Variables	M	SD	SES	Lifestyle	Physical Health	Psychological Health
SES	34.62	15.28	—			
Lifestyle	12.77	4.80	0.56 **	—		
Physical health	7.81	1.82	0.27 **	0.29 **	—	
Psychological health	17.38	3.10	0.12 **	0.21 **	0.36 **	—

Note: ** Significant at level *p* < 0.01.
